# Supplementary material for: Retrospective Inference as a Form of Bounded Rationality, and Its Beneficial Influence on Learning
Source: Front Artif Intell. 2020 Feb 18;3:2. doi: 10.3389/frai.2020.00002 (PMC7861256; doi:10.3389/frai.2020.00002)
Supplement: Supplementary file 1 [file Data_Sheet_1.pdf]

## Mathematical Appendix

### Derivation of recursive state estimation

To derive Equation 3, we first use the product rule of probability to expand

$$\begin{aligned} p(\mathbf{x}_{1:T} \mid \mathbf{o}_{1:T}, \mathbf{x}_0, \boldsymbol{\theta}) &= p(\mathbf{x}_T \mid \mathbf{o}_{1:T}, \mathbf{x}_{0:T-1}, \boldsymbol{\theta})\, p(\mathbf{x}_{T-1} \mid \mathbf{o}_{1:T}, \mathbf{x}_{0:T-2}, \boldsymbol{\theta})\ldots \\ &= \prod_{i=1}^{T} p(\mathbf{x}_i \mid \mathbf{o}_{1:T}, \mathbf{x}_{0:i-1}, \boldsymbol{\theta}). \end{aligned} \quad (1)$$

Given the conditional independence properties expressed in Eq 1, this can be rewritten as

$$\prod_{i=1}^{T} p(\mathbf{x}_i \mid \mathbf{o}_{1:T}, \mathbf{x}_{0:i-1}, \boldsymbol{\theta}) = \prod_{i=1}^{T} p(\mathbf{x}_i \mid \mathbf{o}_{i:T}, \mathbf{x}_{i-1}, \boldsymbol{\theta}) \quad (2)$$

### Derivation of the update equations for the Bayesian HMM

(3)

The log joint for the Bayesian HMM we describe is:

$$\begin{aligned} \ln p(o_{1:T}, x_{1:T}, \mathbf{A}, \mathbf{B} \mid \mathbf{d}, \boldsymbol{\Pi}^a, \boldsymbol{\Pi}^b) &= \sum_{i=1}^{T} \ln p(o_i \mid x_i, \mathbf{B}) + \sum_{i=2}^{T} \ln p(x_i \mid x_{i-1}, \mathbf{A}) \\ &+ \ln p(\mathbf{B} \mid \boldsymbol{\Pi}^b) + \ln p(\mathbf{A} \mid \boldsymbol{\Pi}^a) + \ln p(x_1 \mid \mathbf{d}), \end{aligned} \quad (4)$$

with individual factors given by:

$$\begin{aligned} p(o_i \mid x_i, \mathbf{B}) &= \prod_{k=1}^{M} \prod_{j=1}^{K} B_{jk}^{x_{ij} o_{ik}}, \\ p(\mathbf{A}_{j\bullet}) &= Dir(\mathbf{A}_{j\bullet} \mid \boldsymbol{\Pi}^a_{j\bullet}) = C(\Pi^a_{j\bullet}) \prod_{k=1}^{K} A_{jk}^{\Pi^a_{jk}-1}, \\ p(\mathbf{B}_{j\bullet}) &= Dir(\mathbf{B}_{j\bullet} \mid \boldsymbol{\Pi}^b_{j\bullet}) = C(\Pi^b_{j\bullet}) \prod_{k=1}^{K} B_{jk}^{\Pi^b_{jk}-1}, \\ p(x_1 \mid \mathbf{d}) &= \prod_{j=1}^{K} d_j^{x_{1j}}. \end{aligned} \quad (5)$$

where

$$C(\Pi^a_{j\bullet}) = \frac{\Gamma\left(\sum_{k=1}^{K} \pi^a_{jk}\right)}{\prod_{k=1}^{K} \Gamma(\pi^a_{jk})}, \quad (6)$$

and $\Gamma$ denotes the gamma function.

Since exact inference in this model is intractable, we define a tractable approximating distribution by factorising such that

$$q(x_{1:T}, \mathbf{A}, \mathbf{B}) = q(x_{1:T})\, q(\mathbf{A})\, q(\mathbf{B}) \quad (7)$$

The optimal solution for $q^*\left(x_{1:T}\right)$ is given by:

$$\begin{aligned}
\ln q^*\left(x_{1:T}\right) &= \mathbb{E}_{\mathbf{A},\mathbf{B}}\left[\ln p\left(o_{1:T}, x_{1:T}, \mathbf{A}, \mathbf{B} \mid x_0, \mathbf{\Pi}^a, \mathbf{\Pi}^b\right)\right] + \text{const}, \\
&= \mathbb{E}_{\mathbf{A},\mathbf{B}}\left[\sum_{i=1}^{T} \ln p\left(x_i \mid x_{i-1}, \mathbf{A}\right) + \sum_{i=1}^{T} \ln p\left(o_i \mid x_i, \mathbf{B}\right)\right] + \text{const}, \\
&= \mathbb{E}_{\mathbf{A},\mathbf{B}}\left[\sum_{i=1}^{T}\sum_{k=1}^{K}\sum_{j=1}^{K} x_{i-1j} x_{ik} \ln A_{jk} + \sum_{i=1}^{T}\sum_{k=1}^{M}\sum_{j=1}^{K} x_{ij} o_{ik} \ln B_{jk}\right] + \text{const}, \\
&\propto \sum_{i=1}^{T}\sum_{k=1}^{K}\sum_{j=1}^{K} x_{i-1j} x_{ik} \ln \widehat{A}_{jk} + \sum_{i=1}^{T}\sum_{k=1}^{M}\sum_{j=1}^{K} x_{ij} o_{tk} \ln \widehat{B}_{jk} \\
\ln \widehat{A}_{jk} &\equiv \mathbb{E}\left[\ln A_{jk}\right] = \psi\left(\Pi^a_{jk}\right) - \psi\left(\sum \mathbf{\Pi}^a_{j,\bullet}\right), \\
\ln \widehat{B}_{jk} &\equiv \mathbb{E}\left[\ln B_{jk}\right] = \psi\left(\Pi^b_{jk}\right) - \psi\left(\sum \mathbf{\Pi}^b_{j,\bullet}\right).
\end{aligned} \tag{8}$$

This involves evaluating the entire joint conditional probability distribution over $x_{1:T}$, which as discussed in the main text rapidly becomes computationally infeasible, and is often unnecessary. However, to infer on the optimal marginal distribution over states, we simply need to perform smoothing using the matrices $\widehat{\mathbf{A}}$ and $\widehat{\mathbf{B}}$. (These are not true transition and observation matrices since the rows do not sum to one. However, the relevant normalisation constants are estimated and applied automatically during smoothing) We can then use the results of smoothing to calculate the joint distributions $\xi\left(x_{i-1}, x_i\right)$. If we now define a set of sufficient statistics:

$$\begin{aligned}
\ln \widehat{\mathbf{x}}_i &\equiv \mathbb{E}_{\mathbf{A},\mathbf{B}}\left[\ln p\left(x_i \mid o_{1:T}, \mathbf{d}, \mathbf{A}, \mathbf{B}\right)\right] = \ln \mathbb{E}_{\mathbf{A},\mathbf{B}}\left[\gamma\left(x_i\right)\right] \\
&\propto \left((\widehat{\mathbf{B}} o_i) \circ \left(\widehat{\mathbf{A}}^{\mathrm{T}} \alpha\left(x_{i-1}\right)\right)\right)\left(\widehat{\mathbf{A}}\left(\beta\left(x_{i+1}\right) \circ \left(\widehat{\mathbf{B}} o_{i+1}\right)\right)\right) \\
\ln \widehat{\mathbf{M}}_i &\equiv \mathbb{E}_{\mathbf{A},\mathbf{B}}\left[\ln p\left(x_{i-1}, x_i \mid o_{1:T}, \mathbf{d}, \mathbf{A}, \mathbf{B}\right)\right] = \ln \mathbb{E}_{\mathbf{A},\mathbf{B}}\left[\xi\left(x_{i-1}, x_i\right)\right] \\
&\propto \widehat{\mathbf{A}} \circ \left(\alpha\left(x_{i-1}\right)\left(\left(\widehat{\mathbf{B}} o_i\right) \circ \beta\left(x_i\right)\right)^{\mathrm{T}}\right).
\end{aligned} \tag{9}$$

it is easy to find the optimal solutions for $q^*\left(\mathbf{A}\right)$ and $q^*\left(\mathbf{B}\right)$.

$$\begin{aligned}
\ln q^*\left(\mathbf{A}\right) &= \mathbb{E}_{x_{1:T},\mathbf{B}}\left[\ln p\left(o_{1:T}, x_{1:T}, \mathbf{A}, \mathbf{B} \mid \mathbf{d}, \mathbf{\Pi}^a, \mathbf{\Pi}^b\right)\right] + \text{const}, \\
&= \mathbb{E}_{x_{1:T},\mathbf{B}}\left[\sum_{i=2}^{T} \ln p\left(x_i \mid x_{i-1}, \mathbf{A}\right) + \ln p\left(\mathbf{A} \mid \mathbf{\Pi}^a\right)\right] + \text{const}, \\
&\propto \sum_{i=2}^{T}\sum_{k=1}^{K}\sum_{j=1}^{K} \widehat{M}_{ijk} \ln A_{jk} + \sum_{k=1}^{K}\sum_{j=1}^{K}\left(\Pi^{a0}_{jk} - 1\right)\ln A_{jk}.
\end{aligned} \tag{10}$$

Thus by inspection the optimised factor is:

$$\begin{aligned}
q^*\left(\mathbf{A}\right) &= \prod_{j=1}^{K} \mathrm{Dir}\left(\mathbf{A}_{j,\bullet} \mid \mathbf{\Pi}^a_{j,\bullet}\right), \\
\mathbf{\Pi}^a &= \mathbf{\Pi}^{a0} + \sum_{i=2}^{T} \mathbf{M}_i.
\end{aligned} \tag{11}$$

Similarly

$$
\begin{aligned}
\ln q^*(\mathbf{B}) &= \mathbb{E}_{x_{1:T},\mathbf{A}}\left[\ln p\left(o_{1:T}, x_{1:T}, \mathbf{A}, \mathbf{B} \mid \mathbf{d}, \mathbf{\Pi}^a, \mathbf{\Pi}^b\right)\right] + \text{const}, \\
&= \mathbb{E}_{x_{1:T},\mathbf{A}}\left[\sum_{i=1}^{T} \ln p\left(o_i \mid x_i, \mathbf{B}\right) + \ln p\left(\mathbf{B} \mid \mathbf{\Pi}^b\right)\right] + \text{const}, \\
&\propto \sum_{i=1}^{T}\sum_{k=1}^{M}\sum_{j=1}^{K} \widehat{x}_{ij} o_{ik} \ln B_{jk} + \sum_{k=1}^{M}\sum_{j=1}^{K}\left(\Pi_{jk}^{b0} - 1\right)\ln B_{jk}, \qquad (12) \\
q^*(\mathbf{B}) &= \prod_{j=1}^{K} \text{Dir}\left(\mathbf{B}_{j,\bullet} \mid \mathbf{\Pi}_{j,\bullet}^{b}\right), \\
\mathbf{\Pi}^b &= \mathbf{\Pi}^{b0} + \sum_{i=1}^{T} \widehat{\mathbf{x}}_{ij} \mathbf{o}_{ik}^{\mathbf{T}}.
\end{aligned}
$$

Thus we can perform approximate inference simply by iteratively evaluating the update equations for each factor.

For the probabilistic reversal model, we enforce symmetry between the rows of $\mathbf{A}$, and make use of a single Dirichlet prior that has two concentration parameters (in other words, a beta distribution).

$$
\begin{aligned}
p\left(x_i \mid x_{i-1}, \mathbf{A}\right) &= \prod_{j=1}^{2} A_{1,1}^{x_{i-1,j} x_{i,3-j}} A_{1,2}^{x_{i-1,j} x_{ij}}, \\
p\left(\mathbf{A}_{1\bullet} \mid \mathbf{\Pi}^a\right) &= \text{Dir}\left(\mathbf{A}_{1\bullet} \mid \mathbf{\Pi}^a\right), \\
p\left(\mathbf{A}_{1j} \mid \mathbf{\Pi}^a\right) &= p\left(\mathbf{A}_{2,3-j} \mid \mathbf{\Pi}^a\right), \\
\mathbf{\Pi}^a &= \mathbf{\Pi}^{a0} + \left[\sum_{i=2}^{T}\sum_{j=1}^{2} \widehat{M}_{ij3-j} \quad \sum_{i=2}^{T}\sum_{j=1}^{2} \widehat{M}_{ijj}\right], \\
\mathbf{A}_{2\bullet} &= \left[A_{1,2} \quad A_{1,1}\right].
\end{aligned}
$$
